# Anti-biofilm activities of coumarin as quorum sensing inhibitor for *Porphyromonas gingivalis*

**DOI:** 10.1080/20002297.2022.2055523

**Published:** 2022-03-29

**Authors:** Zhiyan He, Wei Jiang, Yiting Jiang, Jiachen Dong, Zhongchen Song, Jianrong Xu, Wei Zhou

**Affiliations:** a Laboratory of Oral Microbiota and Systemic Diseases, Shanghai Ninth People’s Hospital, Shanghai Jiao Tong University School of Medicine; College of Stomatology, Shanghai Jiao Tong University; National Center for Stomatology; National Clinical Research Center for Oral Diseases; Shanghai Key Laboratory of Stomatology, Shanghai, ‎ China; b Department of Endodontics and Operative Dentistry, Shanghai Ninth People’s Hospital, Shanghai Jiao Tong University School of Medicine; College of Stomatology, Shanghai Jiao Tong University; National Center for Stomatology; National Clinical Research Center for Oral Diseases; Shanghai Key Laboratory of Stomatology, Shanghai, China; c Department of Periodontology, Shanghai Ninth People’s Hospital, Shanghai Jiao Tong University School of Medicine; College of Stomatology, Shanghai Jiao Tong University; National Center for Stomatology; National Clinical Research Center for Oral Diseases; Shanghai Key Laboratory of Stomatology, Shanghai, China; d Academy of Integrative Medicine, Shanghai University of Traditional Chinese Medicine ; Shanghai, China; e Department of Pharmacology and Chemical Biology, Shanghai Jiao Tong University School of Medicine, Shanghai, China

**Keywords:** *Porphyromas gingivalis*, coumarin, biofilm, quorum sensing inhibitor, periodontitis

## Abstract

*Porphyromonas gingivalis* is a keystone pathogen in periodontitis, a biofilm-mediated infection disease. This research aimed to investigate the effect of coumarin on *P. gingivalis* biofilm formation. We detected the antimicrobial effect on *P. gingivalis* planktonic growth, observed membrane structure and morphological change by TEM, and quantified membrane permeability by calcein-AM staining. The cell surface hydrophobicity, aggregation, and attachment were assessed. We also investigated different sub-MIC concentrations of coumarin on biofilm formation, and observed biofilm structureby confocal laser scanning microscopy. The biofilm-related gene expression was evaluated using real-time PCR. The results showed that coumarin inhibited *P. gingivalis* growth and damaged the cell morphology above 400 μM concentration. Coumarin did not affect cell surface hydrophobicity, aggregation, attachment, and the early stage of biofilm formation at sub-MIC concentrations. Still, it exhibited anti-biofilm effects for the late-stage and pre-formed biofilms dispersion. The biofilms after coumarin treatment became interspersed, and biofilm-related gene expression was downregulated. Coumarin also inhibited AI-2 activity and interacted with the HmuY protein by molecular docking analysis. Our research demonstrated that coumarin inhibited *P. gingivalis* biofilm formation through a quorum sensing system.

## Introduction

Periodontitis, a chronic inflammatory disease that leads to irreversible and progressive destruction of periodontal tissues (gingival, periodontal ligament, and alveolar bone) surrounding the teeth and ultimately tooth loss, is induced by dysbiotic microbial biofilm [1,2]. It is one of the most widespread oral diseases, with an overall occurrence of 11.2%, and around 743 million individuals suffer from this disease [3]. Recent reports have revealed that it is also associated with systemic diseases, such as cardiovascular disease, rheumatoid arthritis, diabetes, Alzheimer’s disease, and pregnancy complications [4]. As a keystone pathogen in periodontitis, *Porphyromonas gingivalis* is a Gram-negative, rod-shaped, black-pigmented anaerobic bacterium. *P. gingivalis* can colonize in the subgingival pocket and be incorporated into a subgingival biofilm that initiates periodontitis [5,6]. As a late colonizer of biofilm, *P. gingivalis* adheres to an early colonizer such as *Streptococcus gordonii* and late colonizers such as *Fusobacterium nucleatum* and *Treponema denticola*, as well as to host tissues. When *P. gingivalis* interacts with other oral bacteria and host tissues, several *P. gingivalis* virulence factors, including the capsular polysaccharide, the major fimbrillin FimA, and the minor fimbrillin Mfa1, contribute to biofilm formation [7,8]. These virulence factors produced by *P. gingivalis* evade the host immune defense system and have destructive effects on host periodontal tissues [9]. *P. gingivalis* can also disrupt the balance between the resident microbiota and develop an environment that benefits its own and other pathogens’ continued persistence, thereby modifying the immune response to impair host immune surveillance [10].

Biofilms are formed by microorganisms attached to a substratum and are composed of extracellular polysaccharides (EPS), proteins, lipids, and extracellular DNA [11]. Compared to planktonic bacteria, biofilm communities display specific properties such as being highly resistant to antimicrobial tolerance and better adapted to external environments [12]. Therefore, strategies focusing on eradicating the biofilm phenotype to avoid antimicrobial resistance have recently attracted much attention. Quorum sensing (QS) signals play an essential role in the biofilm development and dispersal [13]. QS is a well-known cell–cell communication system where bacteria produce and respond to signaling molecules known as autoinducers, sense the population density, and coordinate inter- and intra-population behaviour [14]. The bacterial autoinducers as signaling molecules in QS are autoinducer-1 (AI-1, acyl-homoserine lactones), autoinducing peptides (AIPs), and autoinducer-2 (AI-2, furanosyl borate diester) for cell-to-cell communications. Gram-negative bacteria employ acyl-homoserine lactones, whereas Gram-positive bacteria produce autoinducing peptides. Autoinducer-2, produced by the enzyme S-ribosylhomocysteine lyase (LuxS), is used for intra- and inter-species communication in both Gram-positive and Gram-negative bacteria [15–17]. As a new kind of potential antibiotic substitute, QS inhibitors have gained attention for inhibiting bacterial pathogenesis and not inducing antibiotic resistance. Hence, disrupting the QS process, including the application of quorum sensing inhibitors, is a critical way to control biofilm infections [18].

As a promising heterocyclic molecular framework, coumarin consists of a benzene ring joined to a pyrone ring and is found in a wide variety of plant sources, including beans, sweet clover, cinnamon oil and lavender [19]. It has a wide range of broad pharmacological properties, such as anticancer, anti-inflammatory, antimicrobial, antioxidant, and anticoagulant activities [20,21]. Zeng et al. first showed that coumarin had QS and biofilm inhibitory activities as a QS inhibitor through docking analysis against the *Agrobacterium tumefaciens* QS transcriptional activator protein TraR [22]. Further research found that coumarin exhibited a potent inhibitory effect against QS systems, including those of *Pseudomonas aeruginosa* and *Escherichia coli* O157:H7 [23,24].

However, to the best of our knowledge, there was no previous study on the connection between the QS inhibitor coumarin and biofilm formation of *P. gingivalis*. Therefore, this study aimed to investigate the effect of coumarin on *P. gingivalis* biofilm formation. This article focused on biofilm-related parameters, different stages of biofilm formation, biofilm structure, and QS-related gene expression. The regulatory mechanism of this system was also analysed by AI-2 activity and molecular docking.

## Materials and methods

### Bacterial strain and growth condition

The *P. gingivalis* ATCC33277 strain (type strain) was provided by Laboratory of Oral Microbiota and Systemic Diseases, Shanghai Ninth People’s Hospital, Shanghai Jiao Tong University School of Medicine and grown in Brain Heart Infusion Broth (BHI; Difco Laboratories, Sparks, MD) supplemented with hemin (5 μg/mL), vitamin K (0.5 μg/mL) at 37°C under anaerobic conditions (80% N_2_, 10% CO_2_, and 10% H_2_).

### Growth curve assay

**Figure 1.**
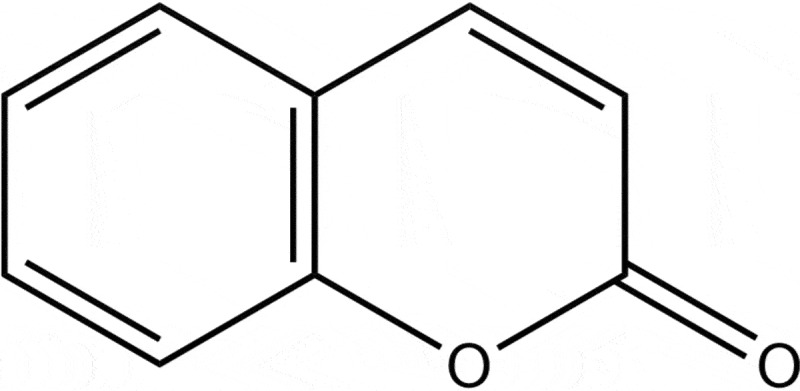
Chemical structures of coumarin.

The coumarin (C_9_H_6_O_2_, Figure 1) was purchased from Sigma-Aldrich (purity ≥ 98%) and dissolved in dimethyl sulfoxide as the stock solution. The various concentrations of coumarin (0, 25, 50, 100, 200, 400, 800, 1,600, and 3,200 μM) were diluted from this stock solution. The *P. gingivalis* strain was used to anaerobically inoculate a fresh BHI culture with different concentrations of coumarin at 37°C anaerobically. The optical density at 600 nm (OD_600 nm_) was measured by a spectrophotometer (UV1601, Shimadzu, Japan) every 6 h intervals throughout incubation. The experiment was replicated three times with triplicate samples at each time point. The minimal inhibitory concentration (MIC) was the lowest concentration of coumarin that inhibited visible bacterial growth. The minimal bactericidal concentration (MBC) was the lowest concentration that yielded no colony growth on blood agar plates.

### Transmission electron microscopy

The *P. gingivalis* cells with different concentrations of coumarin were cultured at 37°C anaerobically for 4 h. The cell pellets were washed with PBS and fixed with 2.5% glutaraldehyde at 4°C. After exposing to 2% osmium tetroxide for 2 h, the cells were dehydrated in a series of ethanol (30%, 50%, 70%, 85%, 95%, 100% and 100%) and dried in acetone solutions three times (50%, 100% and 100%) for 15 min each. Finally, the samples were embedded in resin blocks, cut into ultrathin (70-nm) sections, and stained with uranyl acetate and lead citrate. Images of morphological changes in *P. gingivalis* cells were observed by transmission electron microscopy (TEM, FEI Talos L120C).

### Membrane permeability assay

The membrane permeabilisation of bacterial cells was quantified by a calcein leakage assay using a flow cytometer. The bacteria were treated with different concentrations of coumarin at 37°C using the same process as above. After 4 h treatment, the bacteria were stained with calcein-AM (Thermo Fisher Scientific) according to the manufacturer’s instructions in the dark. Cell fluorescence was collected by a flow cytometer (Agilent ACEA Novocyte 2060 R) at an excitation wavelength of 488 nm and an emission wavelength of 517 nm. All experiments were repeated three times to verify the results.

### Bacterial aggregation assay

The aggregation assay was performed as previously described with minor modifications [25]. Briefly, an overnight *P. gingivalis* suspension was harvested by centrifugation at 12,000 × g for 30 s, washed twice with PBS, and resuspended in the same buffer. Bacterial suspensions were adjusted to an optical density of approximately 1.0 at OD_600 nm_ by using a spectrophotometer. The initial OD_600 nm_ was recorded and the samples with different concentrations of coumarin as above were incubated for 2 h. The percentage of aggregation was calculated by the following equation: Aggregation rate = (OD_Initial_ − OD_2 h_)/(OD_Initial –_ OD_Blank_) × 100%. All experiments were repeated three times to verify the results.

### Hydrophobicity assay

The cell surface hydrophobicity of *P. gingivalis* was determined by measuring adherence to n-hexadecane [25]. Briefly, bacterial cells cultured in BHI with different concentrations of coumarin were washed twice in phosphate urea magnesium buffer (PUM containing 0.115 M K_2_HPO_4_, 0.05 M KH_2_PO_4_, 0.03 M urea, 0.8 mM MgSO_4_ · 7H_2_O; pH 7.2) by centrifugation at 10,000 × g for 5 min and re-suspended in the same buffer. Bacterial suspensions were adjusted to an optical density of 0.6 at OD_550 nm_. Then, 0.4 mL of hexadecane (Sigma) was added to 2 mL of the cell suspensions in tubes. This mixture was vortexed for 60 s, and then incubated for 15 min at room temperature. The OD_550 nm_ of the aqueous phase was measured, and the hydrophobic activity was calculated as follows: [(OD_550 nm_ before mixing) – (OD_550 nm_ after mixing)]/(OD_550 nm_ before mixing) × 100. All experiments were repeated three times to verify the results.

### Attachment assay

The attachment assay was performed as previously described with modifications [26]. Bacteria were harvested by centrifugation and resuspended in sterile PBS. Bacteria at approximately 10^8^ CFU/mL

cell density were incubated at 37°C in a 96-well polystyrene plate for 90 min. Subsequently, the plate was washed with PBS to remove nonadherent cells and stained with 0.1% (w/v) crystal violet. Wells were rinsed with distilled water, dried, and 95% ethanol was added to detect OD _550 nm_ values. All experiments were repeated three times to verify the results.

### Biofilm formation assay

The effect of coumarin on *P. gingivalis* biofilm formation was examined by the 3-(4,5-dimethylthiazolyl-2)-2,5-diphenyltetrazoliumbromide (MTT) and crystal violet method with minor modification [25,27]. A 200 μL aliquot of *P. gingivalis* suspension was grown in BHI supplemented with different concentrations of coumarin for different times (16 and 48 h) at 37°C in 96-well polystyrene plates. The culture supernatant was removed and washed three times with sterile PBS. MTT solution (0.5 mg/mL in sterile PBS) was placed in each well, and the plates were incubated for 3 h in a dark place at 37°C. Following incubation, the MTT solution was gently aspirated from each well, and 100 μL of lysing solution was added to dissolve the formazan crystals. The OD values were recorded at a wavelength of 590 nm by a microplate reader. The biofilm biomass was determined by the crystal violet staining method as in the above attachment assay. All experiments were performed in triplicate with at least three replicates.

### Dispersal of pre-formed biofilms

Bacterial biofilms were grown in 96-well polystyrene plates as described above for 48 h at 37°C to determine whether coumarin could disperse pre-formed biofilms. The biofilm medium was replaced with a fresh medium containing different concentrations of coumarin and incubated for another 24 h at 37°C. Quantification of the biofilm’s metabolic activity and biomass in the 96-well plates were determined by the MTT and crystal violet methods as described above. All experiments were performed in triplicate with at least three replicates.

### Biofilm structure

The structure of *P. gingivalis* biofilms, grown as described in the above assay of biofilm formation and dispersion in the presence or absence of coumarin, was observed by a confocal laser scanning microscope (CLSM). The biofilms formed on the glass-bottom chamber slides were treated with L-7012 LIVE/DEAD BacLight TM bacterial cells (Molecular Probes Inc., Eugene, OR) containing SYTO 9 dye and propidium iodide according to the manufacturer’s instructions. A confocal laser scanning microscope (Leica TCS SP2, Leica microsystems, Germany) was used to record image stacks in five random locations. Five confocal data sets were recorded at 40 × magnification with a numerical aperture of 1.25. In each experiment, the exciting laser intensity, background level, contrast, and electronic zoom were maintained at the same level.

### RNA extraction, reverse transcription and real-time polymerase chain reaction (RT-PCR)

The biofilms grown as described in the above assay of biofilm formation and dispersion in presence or absence of coumarin were harvested and resuspended in TRIzol reagent (Sigma-Aldrich). Total RNA extraction was performed according to the manufacturer’ instructions. Purified RNA was dissolved in 20 μL DEPC-treated water and stored at −80°C until required for cDNA labeling. Reverse transcription was performed by a cDNA synthesis kit (Takara) to generate cDNA. The cDNA samples were stored at −20°C until further use.

The resulting cDNA and negative control were amplified by the Roche LightCycler 480 real-time PCR detection system (Roche, Basel, Switzerland). The reaction mixture (20 μL) contained 10 μL SYBR Premix Ex Taq II (2 ×; Takara), 5 μL template cDNA, 0.4 μL of the appropriate forward and reverse PCR primers and 4.2 μL of sterile distilled water. The PCR conditions included initial denaturation at 98°C for 5 min, followed by a 40-cycle amplification consisting of denaturation at 98°C for 15 s, annealing at 60°C for 15 s, and extension at 72°C for 30 s. We chose the eight biofilm-related genes listed in Table 1 in this study [8,25,28]. The *16S rRNA* gene was used as the housekeeping amplicon. Each assay was performed with at least three independent RNA samples, and fold changes of the expression levels were analyzed using the ΔΔCt method.

**Table 1.** Nucleotide sequences of primers used in this study

Gene*	Accession no. (GenBank)	Description	Primer sequence (5’-3’)	
			Forward	Reverse
*16S rRNA*	PGN_0620	normalizing internal standard	TGTAGATGACTGATGGTGAAA	ACTGTTAGCAACTACCGATGT
*hagB*	PGN_1904	hemagglutinin protein HagB	TGTCGCACGGCAAATATCGCTAAAC	CTGGCTGTCCTCGTCGAAAGCATAC
*kgp*	PGN_1728	lysine-specifc cysteine proteinase Kgp	AGGAACGACAAACGCCTCTA	GTCACCAACCAAAGCCAAGA
*rgpA*	PGN_1970	arginine-specifc cysteine proteinase RgpA	CACCGAAGTTCAAACCCCTA	GAGGGTGCAATCAGGACATT
*rgpB*	PGN_1466	arginine-specifc cysteine proteinase RgpB	GCTCGGTCAGGCTCTTTGTA	GGGTAAGCAGATTGGCGATT
*vimA*	PGN_1056	virulence modulating gene A	TCGCGTAGTCTGAGAGTAACCTT	GGTATAAACGAAGACAGCACGAC
*mfa1*	PGN_0287	minor fimbrial antigen Mfa1	ACTTCTCCCGATTCATGGTG	GGATTCGGGTCAGGGTTATT
*luxS*	PGN_1474	S-ribosylhomocysteine lyase	GAATGAAAGAGCCCAATCG	GTAATCGCCTCGCATCAG

### Quorum sensing AI-2 inhibition assay

*P. gingivalis* was cultured overnight at 37°C under anaerobic conditions with coumarin at various concentrations: 0, 25, 50, 100, and 200 µM. A *P. gingivalis* supernatant was obtained by centrifugation at 10,000 × g for 10 min. AI-2 in the supernatant was detected using the biosensor *Vibrio harveyi* BB170 as previously described with minor modifications [29]. The *V. harveyi* BB170 strain was grown at 30°C in autoinducer bioassay (AB) medium overnight and diluted 1:5,000 into fresh AB medium. Then, 180 µL of the mixture and 20 µL of the supernatant were added to 96-well white plates (Nunc). Bioluminescence of the AI-2 biosynthesis intensity was measured using a multimode plate reader (VCTOR Nivo, PerkinElmer). The AI-2 level was expressed as relative activity (%) based on the AI-2 value of the *V. harveyi* BB170 and *P. gingivalis* supernatant without coumarin. All experiments were performed in triplicate with at least three replicates.

### Molecular docking

The compound coumarin was prepared with the LigPrep module from Schrödinger 2016.1. We used Glide in the Schrodinger 2016.1 standard precision (SP) method for the molecular docking experiment [30]. After protein preparation, the grid was generated by setting the residues around the co-crystallised heme as center and default parameters were applied. Under the OPLS3 force field, the ligand was docked to the *P. gingivalis* heme-binding protein HmuY X-ray structure in the apo state (PDB ID: 6EWM).

### Statistical analysis

All data were presented as means ± SD. One-way analysis of variance (ANOVA) with Dunnett’s post hoc test was used to calculate the significance by SPSS software (SPSS 15.0 software, USA). *P*-values of < 0.05 were considered statistically significant.

## RESULTS

### Antibacterial activity of coumarin on planktonic cell growth

**Figure 2.**
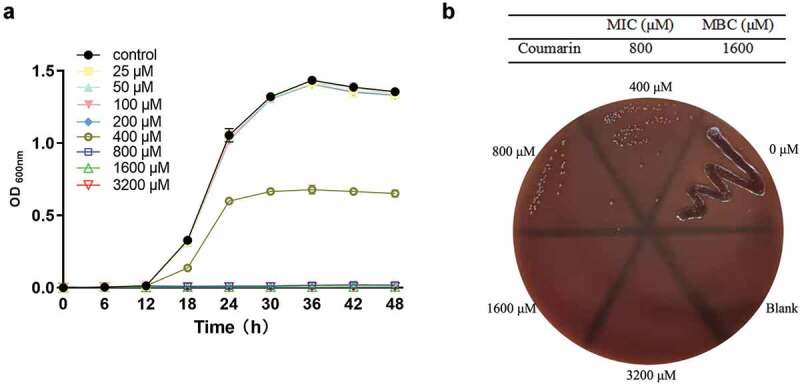
Antibacterial activity of coumarin on *P. gingivalis* planktonic cell growth. (**A**) Growth curve, (**B**) MIC and MBC.

The antibacterial activity of coumarin against *P. gingivalis* planktonic cell growth was determined by the growth rate with different concentrations of coumarin over a time course. The bacterial growth curve is divided into the lag phase (0–12 h), the logarithmic growth phase (12–36 h), and the stationary phase (after 36 h) and a gradually decreasing growth rate (Figure 2A). The results showed no obvious difference in the growth rate below 200 μM coumarin, and 400 μM coumarin led to a 50% reduction compared with the control group. Bacterial growth was almost completely inhibited by coumarin at 800 μM, which was recorded as MIC. The MBC values of coumarin were 1,600 μM (Figure 2B).

### Observation of morphological changes

**Figure 3.**
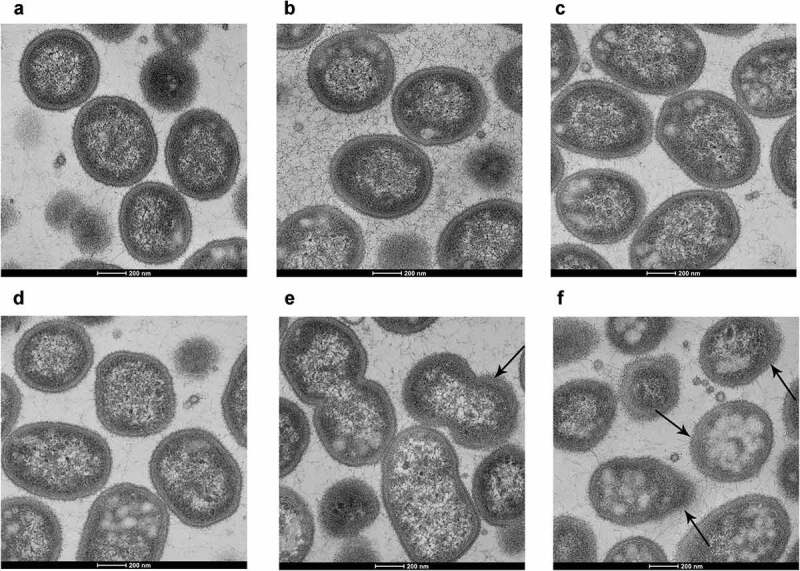
TEM images of *P. gingivalis* treated with different concentrations of coumarin. (**A**) 0 μM, (**B**) 50 μM, (**C**) 100 μM, (**D**) 200 μM, (**E**) 400 μM, (**F**) 800 μM. Bar = 200 nm.

TEM was used to observe the morphological changes of *P. gingivalis* treated with different concentrations of coumarin. The results showed that cell membrane integrity and cell structure in the control group were clearly intact with an obvious boundary (Figure 3A). There was no dramatic change among the control group and sub-MIC concentrations of the coumarin groups (50–200 μM) (Figure 3B–D). As the concentration of coumarin increased from 400 to 800 μM, the cell membrane and cell structures were damaged with increasing severity, and the cell morphology was imperfect with an unclear boundary (Figure 3E, 3F).

### Coumarin changed membrane permeability

**Figure 4.**
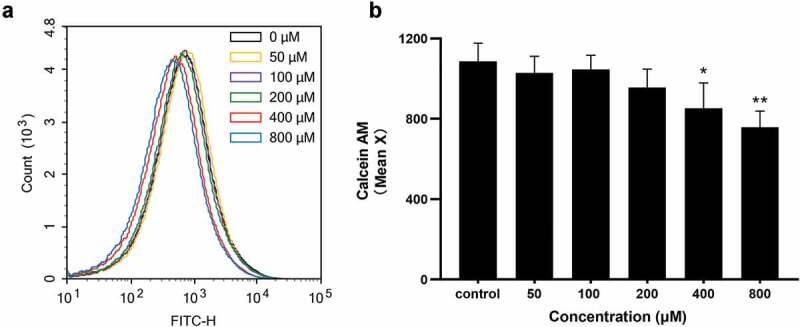
Flow cytometry of calcein AM-stained *P.gingivalis* with coumarin treantment (0–800 µM) (**A**) Representative flow cytometry histograms, (**B**) flow cytometry geometric mean of the fluorescence intensity, Bars denoted by (*) and (**) indicate significant difference at *P*< 0.05, *P*<0.01 by one-way analysis of variance (ANOVA) with Dunnett’s post hoc test.

Bacteria treated with various concentrations of coumarin for 4 h were incubated with calcein-AM to study the effect of coumarin on membrane permeability. Representative flow cytometry histograms are shown in Figure 4A. There was a decrease in calcein-AM fluorescence at 400 and 800 μM coumarin treatment compared to the control group, suggesting that coumarin altered bacterial membrane permeability. However, coumarin below 200 μM did not change geometric mean fluorescence intensity (Figure 4B).

### *Coumarin did not affect* P. gingivalis *aggregation, hydrophobicity, and attachment*

**Figure 5.**
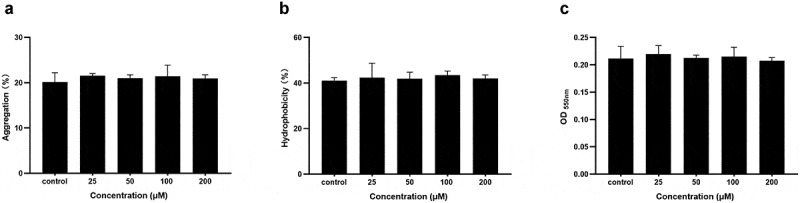
Effect of coumarin on *P. gingivalis* hydrophobicity (**A**), aggregation (**B**) and attachment (**C**) assays.

The aggregation of *P. gingivalis* with different concentrations of coumarin (25, 50, 100, and 200 μM) were 21.57 ± 0.44%, 21.02 ± 0.71%, 21.41 ± 2.49%, and 20.95 ± 0.82%, respectively; which were no obvious differences with that of the control group (21.16 ± 2.05%) (Figure 5A). The hydrophobicity of the bacterial surfaces was determined by measuring the percentage of adherence to hydrocarbons. We compared the *P. gingivalis* surface hydrophobicity rates with different concentrations of coumarin and observed a similar result in aggregation, as shown in Figure 5B. Bacteria adherence to polystyrene plates were quantified, as shown in Figure 5C. There was no significant difference among different concentrations of coumarin. These results indicated that coumarin did not affect the ability of *P. gingivalis* aggregation, hydrophobicity, and attachment.

### Coumarin inhibited different stages of biofilm formation

**Figure 6.**
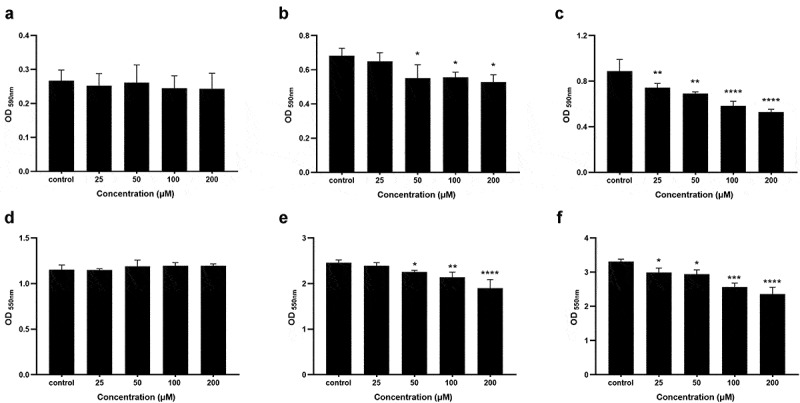
Effect of coumarin on overall biomass and metabolic activity of *P. gingivalis* biofilm. (**A**) metabolic activity of early-stage biofilm formation, (**B**) metabolic activity of late-stage biofilm formation, (**C**) metabolic activity of dispersal of pre-formed biofilm, (**D**) overall biomass of early-stage biofilm formation, (**E**) overall biomass of late-stage biofilm formation, (**F**) overall biomass of dispersal of pre-formed biofilm. Bars denoted by (*), (**), (***) and (****) indicate significant difference at *P* < 0.05, *P* < 0.01, *P*< 0.001, *P* < 0.0001 by one-way analysis of variance (ANOVA) with Dunnett’s post hoc test.

We selected coumarin concentrations below 200 μM to investigate the effect of coumarin on biofilm formation without affecting *P. gingivalis* growth. We studied the effect of coumarin on the metabolic activity of *P. gingivalis* biofilm at different stages by MTT assays. The results showed no significant difference at 16 h among different concentrations of coumarin (Figure 6A). After 48 h of incubation, *P. gingivalis* exhibited OD_590 nm_ values of 0.682 ± 0.044. With increasing concentrations of coumarin, the OD_590 nm_ values decreased from 0.649 ± 0.050 at 25 μM to 0.528 ± 0.042 at 200 μM after incubation for 48 h (Figure 6B). Pre-formed *P. gingivalis* biofilms were used to determine the ability of coumarin to disperse mature biofilms. Biofilms formed on 96-well plates were treated with different concentrations of coumarin in fresh media for 24 h and quantified by the MTT assay. Compared to the controls, an increased biofilm dispersion with increasing concentrations of coumarin was observed in Figure 6C. Treatment with coumarin at 200 μM showed the most substantial biofilm dispersion effect, which could disperse the pre-formed biofilm by approximately 40.4%. Similar results were observed by the crystal violet assay to quantify the overall biomass of biofilms, which were shown in Figure 6D–F.

### Confocal microscopic observation of biofilm structure

**Figure 7.**
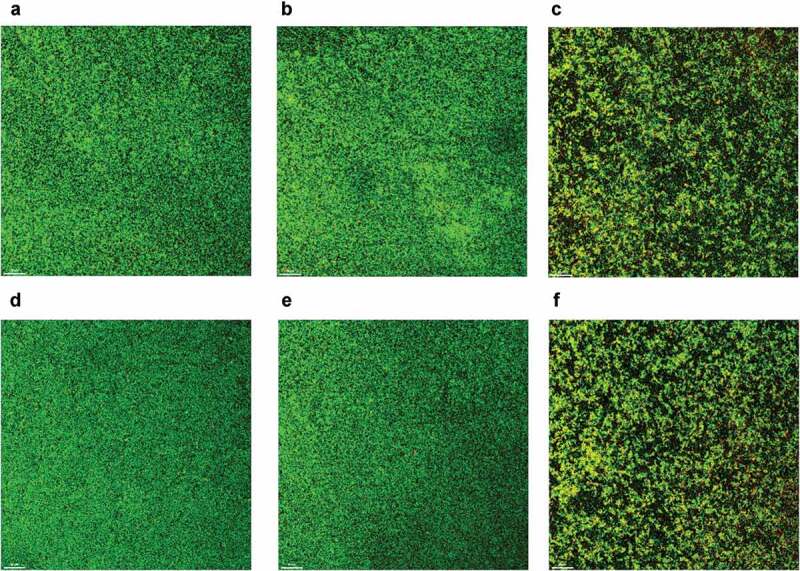
CLSM images of *P. gingivalis* biofilm treated with different concentrations of coumarin. Biofilm formation, (**A**) 0 μM, (**B**) 100 μM, (**C**) 200 μM. Dispersal of pre-formed biofilm, (D) 0 μM, (**E**) 100 μM, **(F**) 200 μM.

The confocal laser scanning microscope was performed to evaluate the effects of coumarin on the biofilm structure after 48 h of incubation. The biofilm of the control group had a uniform distribution with complete coverage of the attached surface (Figure 7A). Following treatment with coumarin, the biofilms appeared highly dispersed and clearly loose (Figure 7B–C). We calculated the proportion of viable (green) cells among all cells, and there were no apparent differences among these groups (data not shown). The structure of pre-formed biofilms treated by coumarin was also observed. As shown in Figure 7D–F, a similar result was found as above after coumarin treatment, and the proportion of viable (green) cells among all cells had no noticeable difference among these groups (data not shown).

### Coumarin inhibited gene expression

**Figure 8.**
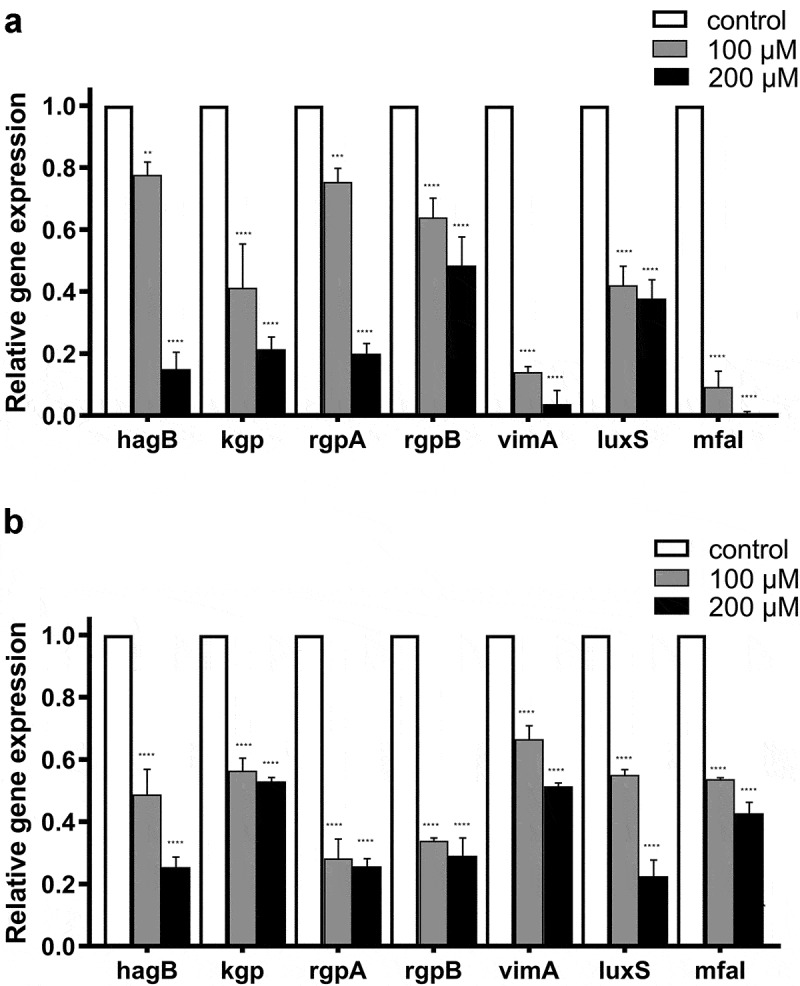
Effect of coumarin on gene expression by real time-PCR. (**A**) Biofilm formation, (**B**) Dispersal of pre-formed biofilm. Bars denoted by (****) indicate significant difference at *P* < 0.0001 by one-way analysis of variance (ANOVA) with Dunnett’s post hoc test.

Real-time PCR analysis was performed to quantify the effect of coumarin on *P. gingivalis* for gaining insight into biofilm-related gene expression. Compared with the control group, all the genes tested (Table 1) were down-regulated in a dose-dependent manner. These genes included *hagB* (involved in hemagglutination); *kgp, rgpA, rgpB* (involved in gingipain); *vimA* (virulence modulating gene A), *luxS* (S-ribosylhomocysteine lyase), and *mfa1* (fimbria major subunit). Among them, the expressions of *mfa1* in the biofilms treated by coumarin after 48 h of incubation were significantly reduced compared to the control group, by about 0.0925-fold at 100 μM and 0.00767-fold at 200 μM, respectively (Figure 8A). We also detected gene expression in pre-formed biofilms treated by coumarin. Transcriptions of *rgpA* at 100 μM and *luxS* at 200 μM were significantly decreased (0.283- and 0.225-fold, respectively) (Figure 8B). The results indicate that coumarin inhibited gene expression in biofilm formation and pre-formed biofilms dispersal.

### Coumarin reduced quorum sensing AI-2 activity

**Figure 9.**
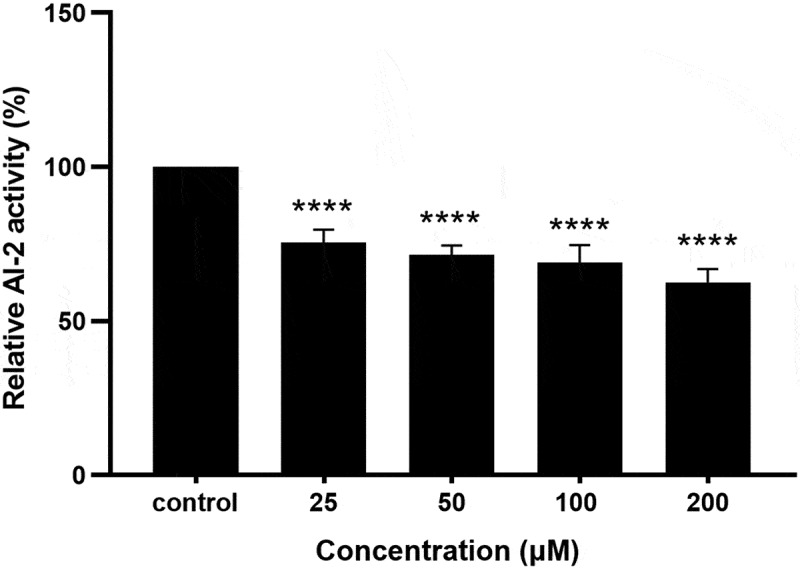
Effect of coumarin on quorum sensing AI-2 inhibition activity. Bars denoted by (****) indicate significant difference at *P* < 0.0001 by one-way analysis of variance (ANOVA) with Dunnett’s post hoc test.

Bioluminescence assays with *V. harveyi* BB170 as a biosensor and *P. gingivalis* supernatants as a measure of LuxS AI-2 activity were used to determine the effect of quorum sensing AI-2 inhibition. As shown in Figure 9, the AI-2 activity inhibitory effects were observed with various concentrations of coumarin. AI-2 inhibition with 200 µM coumarin was significantly reduced compared to the control, while it also exhibited correspondingly lowered bioluminescence activities after coumarin from 25 to 100 μM treatment. These results showed that coumarin could effectively reduce AI-2 activity produced by *P. gingivalis*.

### Binding model analysis

**Figure 10.**
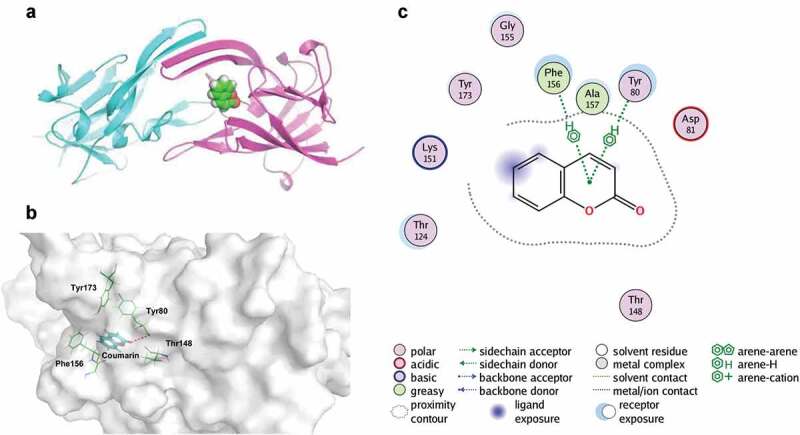
The interaction mode between coumarin and HmuY protein. (**A**) Coumarin was located in the heme binding pocket. (**B**) Coumarin interacted with HmuY. (**C**) The interaction diagram between coumarin and HmuY.

When docked into the *P. gingivalis* heme-binding protein HmuY, coumarin fitted one of the pockets well, as shown in Figure 10A. Two HmuY subunits were coloured in purple and cyan, and coumarin was coloured in lime. In constract to the orthosteric ligand heme, coumarin could not interact with His109 and His141, which interacted with the metal ion of heme. It was observed in Figure 10B that coumarin laid in the pocket consisting of Tyr80, Thr148, Phe156, and Tyr173. The surface of HmuY was presented in white, while the key residues were coloured in green lines. Moreover, there was a polar interaction between Tyr80 and the keto group located at the 2-position of coumarin. In addition, both Tyr80 and Phe156 could interact with the aromatic centre of coumarin via arene-hydrogen bonds, according to the interaction diagram in Figure 10C.

## DISCUSSION

In recent years, the rapid increase in antibiotic resistance has made it imperative to discover and develop novel strategies for controlling microbial infections and bacterial diseases. One of the most promising strategies is targeting QS to prevent biofilms formation and dispersion of existing biofilms. QS systems are essential for regulating the biofilm mode of growth and gene expression related to virulence phenotypes [31,32]. Over the last decades the coumarin class derived from plant extracts have received considerable attention as QS inhibitors and antibiofilm agents [23,24]. Lee et al. found that coumarin and its seven derivatives, including coumarin-3-carboxylic acid, dephnetin, ellagic acid, esculetin, 4-hydroxycoumarin, scopoletin, and umbelliferone (7-hydroxycoumarin), had antibiofilm activity against the *E. coli* O157:H7 strain [23]. Furocoumarins (dihydroxybergamottin and bergamottin) isolated from grapefruit juice inhibited *E. coli* O157:H7 biofilm formation at 72 and 58.3%, respectively, and exhibited strong inhibition of both AI-1 and AI-2 activities in QS systems [33]. Yang et al. examined 18 different coumarins and derivatives for antibacterial and antibiofilm activity against *Ralstonea solanacearum*. Their results found that coumarin and three different hydroxycoumarins (umbelliferone, esculetin, and dephnetin) showed the highest biofilm formation inhibition [34]. Coumarin displayed a broad range of different acylated homoserine lactones (AHLs) inhibition activity, which suggested that it may inhibit native AHL-QS systems in bacterial pathogens [23]. However, to the best of our knowledge, there was no previous study on the connection between the QS inhibitor coumarin and biofilm formation of *P. gingivalis*. Therefore, we would investigate the effect of coumarin on *P. gingivalis* biofilm formation in the present study.

Firstly, we detected the role of coumarin on bacterial growth. Our results showed that coumarin completely inhibited the bacterial growth rate at 800 μM, but the growth curve had no noticeable difference below 200 μM coumarin. Coumarin compounds have antibacterial activity since the coumarin group blocks ATPase activity of the bacterial DNA gyrase in competition with ATP for binding to the B subunit of the enzyme [35]. We also observed the morphological changes by TEM and found that coumarin damaged the cell membrane integrity at 400 and 800 μM. These results were confirmed by membrane permeabilisation assays and were consistent with the previous study [36]. In order to avoid the antibacterial effect, we chose the concentration of coumarin below 200 μM to determine the effect of coumarin on biofilm formation without affecting *P. gingivalis* growth.

The ability of the microbiota to form biofilms would lead to an increase in conventional antibiotics resistance, 10- to 1,000-fold higher than their planktonic mode. For example, the antibiotics such as minocycline and metronidazole could be effective against planktonic *P. gingivalis*, but *P. gingivalis* growth within a biofilm was highly resistant to these antibiotics [37]. Biofilm formation is generally considered a complex process, including initial attachment of the bacterial cells to a surface, followed by growth in multilayered cell clusters, maturing into a complex three-dimensional structure, and finally detachment [38]. Cell surface physicochemical properties, including aggregation ability and surface hydrophobicity, are crucial for bacterial attachment, which is the initial step for biofilm formation [39,40]. Aggregation is considered essential for the development of dental plaque and other mixed-species biofilms and influences the cell mass and architecture of biofilms [41,42]. Cell surface hydrophobicity is a primary parameter for controlling the adhesion of bacteria to the tooth surface. It is associated with bacterial adhesiveness, varying from organism to organism and strain to strain, and is influenced by the growth medium, bacterial age, and bacterial surface structures [43]. In this study, there was no significant difference in *P. gingivalis* initial attachment and its influencing factors (aggregation ability and surface hydrophobicity) with or without coumarin. We also studied the effect of coumarin on the different stages of biofilm formation against *P. gingivalis*. Our results showed that coumarin did not affect the early stage of biofilm formation, consistent with the initial attachment assays. The MTT and crystal violet assays revealed that coumarin below 200 μM disrupted the late stage of biofilm formation and caused the dispersal of pre-formed biofilms. Confocal imaging also confirmed that both kinds of biofilm were thinner and sparser after coumarin treatment. Furthermore, the proportion of viable (green) cells to all cells following coumarin treatment was similar to the proportion without treatment (data not shown). These results suggested that coumarin inhibited biofilm biomass possibly through *P. gingivalis* quorum sensing systems.

Our data showed that all selected biofilm-related genes were downregulated after adding coumarin in the biofilm formation. Among them, the *vimA* (virulence modulating) gene has a multifunctional role in modulating oxidative stress resistance, acetyl-CoA transfer, lipid A synthesis, glycosylation and anchorage of several surface proteins including the gingipains and biofilm-forming capacity [44,45]. The *hagB* gene encodes for hemagglutinin molecules and plays a vital role in mediating attachment to host cells, oral colonization and biofilm formation [46]. Gingipains, the major virulence factors of *P. gingivalis*, can be divided into arginine-specific (Rgp, including RgpA and RgpB) and lysine-specific (Kgp) proteases according to substrate specificity. Gingipains are essential for acquiring nutrients, biofilm formation, and evading of the host defence system [47,48]. Our results showed that coumarin downregulated three gingipain genes consistent with previous studies [49,50]. *P. gingivalis* possesses two distinct types of fimbria, which are filamentous structures that mediate attachment in the oral cavity. One of them is Mfa1 fimbriae which is composed mainly of polymers of Mfa1 proteins. Mfa1 is essential for biofilms formation, including binding to synergistic species in oral biofilms [51,52]. *P. gingivalis* has a LuxS/Autoinducer-2 (AI-2) quorum sensing system, which regulates about 1% of the *P. gingivalis* genome, including biofilm formation, proteinase and hemagglutinin activities [53]. Burgess et al. constructed a *luxS* mutant strain that produced less heamagglutinin and had less protease activity (Rgp and Kgp) than the wild type strain [54]. Our study showed that coumarin reduced the amounts of the biofilm-related regulatory genes at the transcriptional level, ultimately attenuating the biofilm formation ability.

Finally, we investigated the effect of coumarin on the *P. gingivalis* quorum sensing system. Previous studies proved that *P. gingivalis* could produce a signaling molecule(s) to stimulate bioluminescence with the AI-2 biosensor *V. harveyi* BB170 and process an orthologue of LuxS, which exhibited 29% identity with LuxS of *V. harveyi* [54,55]. Thus, *P. gingivalis* has a LuxS/Autoinducer-2 (AI-2) quorum sensing system. LuxS/AI-2 signaling is important for *P. gingivalis* to interact with other species of bacteria in oral biofilms, such as *S. gordonii* [56]. It also regulated hemin acquisition and proteases and stress-related gene expression in *P. gingivalis* itself [57,58]. As a quorum sensing inhibitor, we detected the AI-2 activity by bioluminescence assays of *V. harveyi* BB170 as a biosensor and found that coumarin could inhibit the AI-2 activity effectively. The genome of *P. gingivalis* has many transcriptional regulators, including the LuxR family. A novel transcriptional activator that belonged to the LuxR family was found to regulate the hmu operon in a cell density-dependent manner [59]. HmuY is a heme-binding lipoprotein that plays a major role in *P. gingivalis* heme acquisition systems (Hmu) [60]. Heme is an essential nutrient for *P. gingivalis* to survive, proliferate, and establish infection [60]. Previous studies found that *hmuY* gene expression was quorum sensing dependent, and *P. gingivalis* produced higher levels of HmuY when growing in the biofilm structure [61,62]. Therefore, we chose HmuY as the target protein to reveal anti-QS mechanism of coumarin and confirmed that coumarin bound tightly to HmuY in a molecular docking assay. Therefore, these results proved that coumarin could regulate the *P. gingivalis* quorum sensing system to inhibit biofilm formation.

Oral biofilm, which contains around 700 distinct bacterial species, is more complex than a single species bacterial biofilm. The multispecies biofilm model closely mimicks the complex microbiota of periodontal pathogens in the oral cavity. Therefore, further studies are required to evaluate the effects of coumarin for periodontal multispecies biofilms. In addition, our present results are still far from the clinical therapeutic use of coumarin. Further investigations, such as cell models *in vitro* and animal experiments *in vivo* need to studied.

## Conclusion

Our study demonstrated that coumarin as a quorum sensing inhibitor at sub-MIC concentrations without affecting bacterial growth, inhibited *P. gingivalis* biofilm formation, including biofilm-related parameters, different stages of biofilm formation, biofilm structure, and biofilm-related gene expression. The regulatory mechanism was also analyzed by AI-2 activity and molecular docking. Our study provides new evidence that the natural plant coumarin without inducing antimicrobial resistance might be beneficial in preventing or treating *P. gingivalis* biofilm and periodontal disease.
